# Effects of a semi-rigid ankle brace on ankle joint loading during landing on inclined surfaces

**DOI:** 10.1080/23335432.2018.1481767

**Published:** 2018-07-21

**Authors:** Ilias Theodorakos, Jan Rueterbories, Morten E. Lund, Eric Eils, Michael S. Andersen, Mark De Zee, Uwe G. Kersting

**Affiliations:** a Sport Sciences, Dept. of Health Science and Technology, Aalborg University, Denmark; b Dept. of Mechanical and Manufacturing Engineering, Aalborg University, Denmark; c Institute of Sport and Exercise Sciences, Westfälische Wilhelms-Universität Münster, Germany

**Keywords:** Ankle brace, landing, ankle joint, brace contact pressure, kinematics, inverse dynamics

## Abstract

Ankle bracing is commonly used to prevent ankle sprain occurrences. The present study investigated the effects of a semi-rigid ankle brace on the ankle joint complex during landing on inclined surfaces. Seventeen recreational athletes performed a single leg landing task onto three different surface alignments (everted, neutral, inverted), with and without the brace. Ground reaction forces (GRF), kinematics, and brace pressure were recorded. Six two-way repeated measures MANOVA tested for differences in GRF, talocrural and subtalar kinematics and kinetics. Participants landed with a significantly less plantar flexed (P < 0.001) and more everted (P = 0.001) foot during the braced condition. Although no differences were observed for the joint moments, an increased subtalar compression force (P = 0.009) was observed with the brace. Landing on the inverted surface resulted in significantly higher peak magnitudes of the vertical and the mediolateral GRF and the talocrural inversion moment compared to landing on the neutral surface. Ankle bracing altered ankle kinematics by restricting the ROM of the ankle joint complex. This study confirmed that landing on inverted surfaces may increase the risk for lateral ankle ligaments injuries. The significantly higher subtalar compression force during the brace condition might contribute to overuse injuries.

## 1. Introduction

Ankle sprains are among the most common injuries in sports with ankle ligament injuries constituting about 25% of the injuries occurring in running and jumping sports (Balduini et al. 1987; Bahr and Krosshaug 2005). Landing on irregular surfaces such as on another player’s foot has been identified as a common injury scenario for ankle sprain injuries (Garrick 1977; Wright et al. 2000; Gross and Liu 2003) as, e.g. 87% of ankle sprains among volleyball players were contact injuries (Bahr and Bahr 1997). Ankle bracing is a commonly used intervention to reduce ankle sprain injuries whereas their effectiveness to prevent ankle re-injury occurrences is well documented (Surve et al. 1994; Beynnon et al. 2002), more research is needed to establish their prophylactic role when used by healthy individuals (Dizon and Reyes 2010).

The functionality of an ankle brace and its effects on the ankle kinematics depend on its type and design. Typically, ankle braces are designed to prevent ankle injuries by restricting ankle motion in the frontal plane without interfering with sagittal plane motion. However, there is evidence that semi-rigid braces restrict normal ankle plantarflexion (Siegler et al. 1997). Similar results have been demonstrated in landing trials performed by human participants with rigid braces (McCaw and Cerullo 1999; Cordova et al. 2010). Ankle plantarflexion is important for the attenuation of the ground reaction forces (GRF). Thus, an ankle brace that restricts ankle motion in the frontal plane may have negative effects on ankle and knee loading.

Tilt plates (Eils et al. 2002), cutting maneuvers (Simpson et al. 1999), and landing protocols (Cordova et al. 2010) have been used to test ankle braces under dynamic conditions. A study where landing on inclined surfaces was compared against trap door tests reported an earlier maximum inversion when individuals landed on inclined surfaces (Chen et al. 2012). Based on their results, the authors suggested landing on inclined surfaces as a more realistic scenario than trap door tests for investigating ankle braces and lateral ankle injury mechanisms, without mentioning whether the participants knew the inclination of the surface before performing the landing task (Chen et al. 2012).

Studies investigating brace effects on GRF, ankle kinematics and kinetics during landings vary in their results depending on the type of brace, the landing task, and the testing population. In systematic reviews of two leg landing tasks (Niu et al. 2014) and one also including single leg landing tasks (Niu et al. 2016), it was reported that ankle stabilizers (ankle taping or bracing) increased the peak magnitude value of the vertical GRF. However, the authors stated that the reviews pooled studies employing various types of landing tasks, which may have different GRF features (Niu et al. 2016). Furthermore, the type of the ankle stabilizer and the employed testing populations may also influence the resulting GRF. In addition to this, no differences were reported in the peak magnitudes of the GRF components during single leg landing tasks performed with a semi-rigid ankle brace (Cordova et al. 2010), the ASO lace-up brace (DiStefano et al. 2008), and the Swede-O-Brace lace-up (Hopper et al. 1999) compared to the unbraced condition.

Regarding ankle kinematics, reduced ankle plantarflexion at initial contact (IC) of the foot with the ground and reduced ankle plantarflexion range of motion (ROM) have been reported when participants landed with the ASO lace-up brace (DiStefano et al. 2008; Simpson et al. 2013). Moreover, reduced plantarflexion ROM during the braced condition was reported for a semi-rigid ankle brace (McCaw and Cerullo 1999; Cordova et al. 2010) and for a lace-up ankle brace (Vanwanseele et al. 2014).

Inverse dynamics calculations have been employed to investigate the kinetic effect of ankle braces while the mechanical contribution of the brace itself was not included in such analyses (Venesky et al. 2006; Gardner et al. 2012; Vanwanseele et al. 2014). An increased ankle plantarflexion moment was reported for a lace-up ankle brace (Vanwanseele et al. 2014) whereas a higher ankle eversion moment was observed for the Active Ankle-T2 brace (Venesky et al. 2006). Moreover, the DonJoy Velocity brace significantly reduced the relative ankle work compared to the unbraced condition, but no differences were observed for a hinged brace (Gardner et al. 2012). These studies tested the effectiveness of ankle braces during landing on either neutral or inverted surfaces. None of these studies included a varying inclination of the landing surface as a factor.

The goal of the present study was to investigate whether a semi-rigid ankle brace has an effect on ankle kinematics, resultant joint reaction forces, joint moments, and GRF. Landing trials on inclined surfaces at varying tilt angles were employed to simulate landing on irregular surfaces as may occur during sports participation. We hypothesized no differences in the peak magnitudes of the GRF components, but reductions in the ankle complex kinematics in both the frontal and sagittal planes, which would result in altered ankle joint reaction forces and moments. We based our hypothesis on previous studies showing semi-rigid braces that restrict normal plantarflexion (McCaw and Cerullo 1999; Cordova et al. 2010), but result in similar peak GRF during landing (Cordova et al. 2010). The study by Cordova et al. (2010) was chosen to justify our hypothesis as it employed a similar testing population (male recreational athletes), testing ankle brace type (semi-rigid), and landing task (single leg drop landings). Regarding the inclination factor, we hypothesized differences in the kinematics and kinetics of the ankle joint complex, after IC. The rationale behind our hypothesis is that landing on inclined surface alignments alters foot in/eversion, leading to a different foot positioning, which potentially alters the load transfer within the talocrural and subtalar joints.

## 2. Methods

### 2.1. Experimental data

Twenty healthy males were recruited for the study; however, three of them were excluded from the data analysis due to missing data. The mean age, height and body mass of the 17 remaining participants were 25.7 (4.5) years, 1.80 (0.08) m and 78.3 (6.0) kg, respectively. The participants did not have any prior ankle injuries. An informed consent form was signed by each participant, and the study was approved by the local ethics committee (North Jutland, case number N-20,090,021).

Initially, a reference trial was recorded. Participants were standing upright with both their feet on a force platform (Van Doornik and Sinkjaer 2007). This trial served for the initial estimation of the ankle joints orientations and the body mass of the participants. Next, functional trials were recorded to determine the positions and the orientations of the joints axes via an optimization procedure (Reinbolt et al. 2005). During these trials, participants exercised the respective joint in a wide range of motion, while standing on their non-dominant leg. For the ankle trial, clockwise and anti-clockwise rotations of the foot were recorded while, for the knee trial, knee extensions and flexions were employed. For the hip trial, movements of the testing leg anteriorly, posteriorly and internal, external leg rotations were recorded.

**Figure 1.**
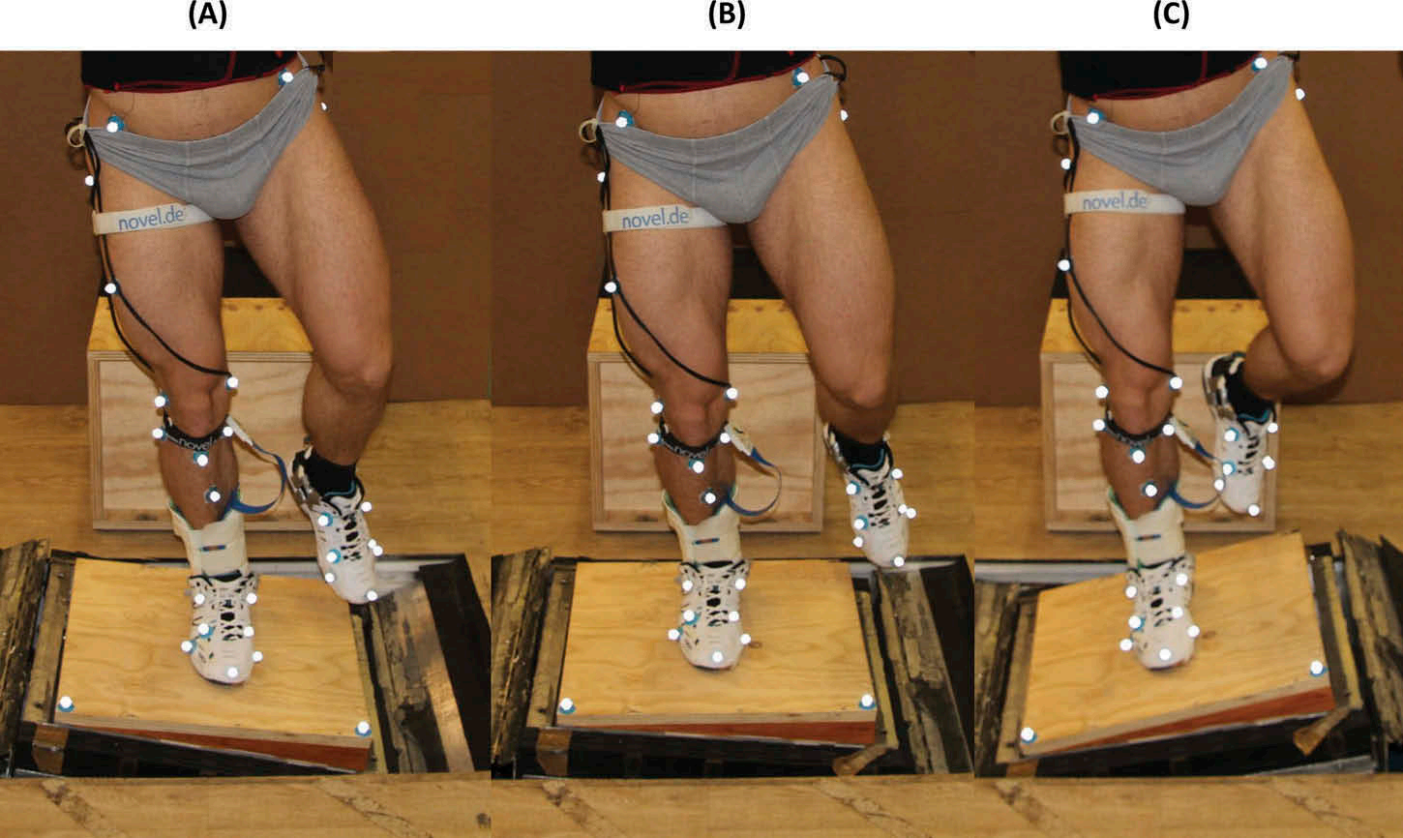
Anterior view of the landing orientation on three different surface alignments. The marker protocol is visible except the heel and the right and left posterior superior iliac spine markers. The malleoli markers were used only at the reference trial. The foot was forced to a different orientation depending on the alignment of the landing surface. The foot had a more everted orientation on the everted alignment (A), whereas it had a more inverted orientation on the inverted alignment (C) compared to the neutral (B)

**Figure 2.**
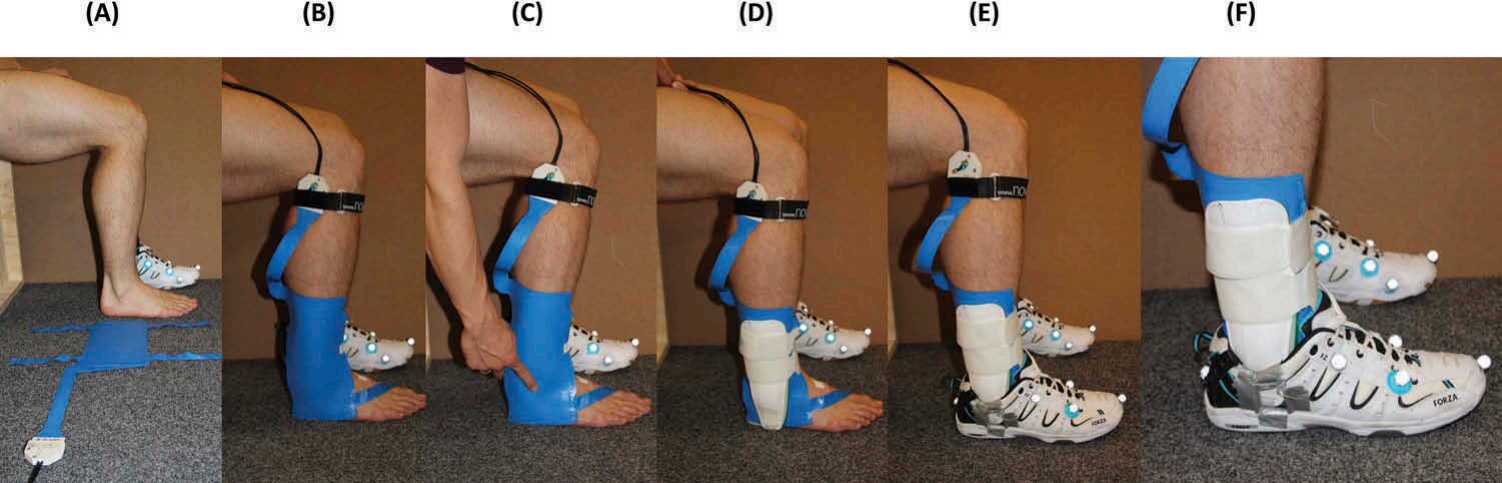
Demonstration of the pressure mat placement on the lateral side of the testing leg. (A) Alignment of pressure mat with the testing leg. (B) Pressure mat placement on the lateral side of the foot and lower leg. (C) Identification of the lateral malleolus position relative to the mat by applying manual force on it. (D) Ankle brace placement. (E) Footwear attachment. (F) Close-up photograph of the testing leg after the placement of the pressure mat and the ankle brace

Subsequently, participants performed single-leg landings by using their dominant leg, which was defined by asking the participants the leg they would use to kick a ball as far as possible. The same method for defining the dominant leg has previously been used by Niu et al. (2011). The dominant leg was selected as the testing leg, because it has been suggested that the dominant ankle joint is at greater injury risk during drop landings compared to the non-dominant and thus, it might produce more conservative conclusions for injury related investigations (Niu et al. 2011). The participants started by standing on a pedestal 40 cm above the ground, with their dominant leg extended over the robotic force platform. At a signal from the principal investigator, the participants pushed off with their non-dominant leg and landed with their dominant foot on a wooden platform, which was rigidly attached on top of the robotic platform (Figure 1). The wooden platform had 5° inverted inclination, which ensured that the robotic platform (range of ± 10°) could produce a greater inversion angle. For each trial, the robotic platform was randomly inclined to produce one out of three landing surface alignments; 5° everted, neutral, and 15° inverted (Figure 1). Measures were taken to ensure the same visual and audible conditions among the different alignment levels. The participants were instructed to look forward during the task, while the starting position of the platform was set at 5° inverted alignment. Following the investigator’s signal, participants started the landing task while the robotic platform was triggered to move. The landing surface alignment was achieved while the participants were airborne. The procedure was performed for two brace conditions: with and without a semi-rigid ankle brace (Aircast Sports Stirrup, Eagle Tor, Derbyshire, England) (Figure 2 D-E). A trial was successful when participants managed to land on the platform with their dominant leg and maintain their balance for at least 2 s. All participants wore the same shoe model to avoid footwear variations (Badminton Shoe FZ809 Olympian Blue, FZ Forza, Denmark).

A force plate (OR6/7, AMTI, Watertown, USA) measured GRF and moments at 4 kHz, while a motion tracking system with eight infrared digital video cameras (Oqus 300 series, Qualisys, Gothenburg, Sweden) captured the motion of the pelvis and the testing extremity at 250 Hz.

Retroreflective, ball-shaped markers were placed on anatomical landmarks of the pelvis and the testing extremity, following the work by Leardini et al. (2007) (Figure 1). The marker protocol was modified to accommodate the needs of the present study (footwear, ankle brace). Markers on the shoe were placed over the first and the fifth metatarsal, over the navicular, the cuboid, and over the second toe, while a hole was made in the shoe in order to place the heel marker. Additional markers were placed on the shank and the thigh. The additional thigh marker was placed on the lower lateral side of the thigh approximately 15 cm from the lateral femoral condyle, while the additional shank marker was placed on the anterior medial side of the tibia approximately 10 cm from the tibial tuberosity.

Contact pressure between the ankle brace and the lower leg was measured by a pressure system (Pliance® pressure mat system, Novel, Munich, Germany) at 100 Hz. Two pressure mats of 16 × 16 sensors with an individual area of 14.13 x 14.13 mm^2^ were placed between leg and brace (Figure 2). The lower edges of the mats were aligned parallel to the ground with the subject standing upright and the foot neutrally aligned. It was ensured that the lower parts of the mats covered the medial and lateral aspects of the rearfoot. Before applying the ankle brace, the pressure mats were secured with tape to ensure that the mat orientation was maintained during the experiment. An offset deduction of the pressure system was performed, in accordance with the manufacturer’s instructions, to account for potential pressure offsets due to the deformation of the mats for each participant. Prior to brace attachment, the relative position of the malleoli on the pressure mats was recorded by manually applying pressure at the respective locations. Each mat was divided into two areas: below and above the malleoli. The center of pressure (COP) expressed with respect to the malleoli locations, and the net force of each area were computed for all the braced trials. A common trigger was used for the force plate, the motion analysis system, and the pressure system in order to synchronize all recordings.

### 2.2. Computational models

#### 2.2.1. Kinematic model

The kinematics of the landing trials were computed by a five-segment stick figure model (Lund et al. 2015), which was developed in the AnyBody Modeling System™ (AMS) v.5.2. (AnyBody Technology A/S, Aalborg, Denmark). It consisted of the pelvis, thigh, shank, the talus, and the foot and it represented the patient-specific topology; joint positions, orientations, anatomical landmarks.

The model was constructed based on the anatomical markers in the reference trial. Following the ISB recommendations (Wu et al., 2002) for calculation of joint angles, local reference frames of the ankle joints were embedded in each of the connected segments. The recommended definitions for the tibia/fibula and calcaneus coordinate systems were employed for the shank and foot segments, respectively (Wu et al., 2002). Since no recommendations for the definition of the talus coordinate system exist, the talus coordinate system was defined to have the same orientation as the tibia/fibula reference frame in the standing reference trial (Suppl). The subtalar and talocrural articulations were modeled as hinge joints and they connected the foot with the talus and the talus with the shank respectively. Subtalar inversion was defined as the positive rotation about the e2 axis of the subtalar joint coordinate system, while the talocrural plantarflexion was defined as the negative rotation about the e1 axis of the talocrural coordinate system (Suppl).

The joint parameters, locations, and directions of the joints were identified using an optimization-based approach. For the ankle, an initial estimation of the joint locations was obtained from the malleoli markers in the reference trial. Then, the ankle functional trial was used to optimize the axes directions of the joints and their local positions as described by Reinbolt et al. (2005), and implemented in AMS by a computational method (Andersen et al. 2010). In this approach, the distance between model markers and recorded markers was minimized using a least squares approach by altering the ankle joint parameters.

**Figure 3.**
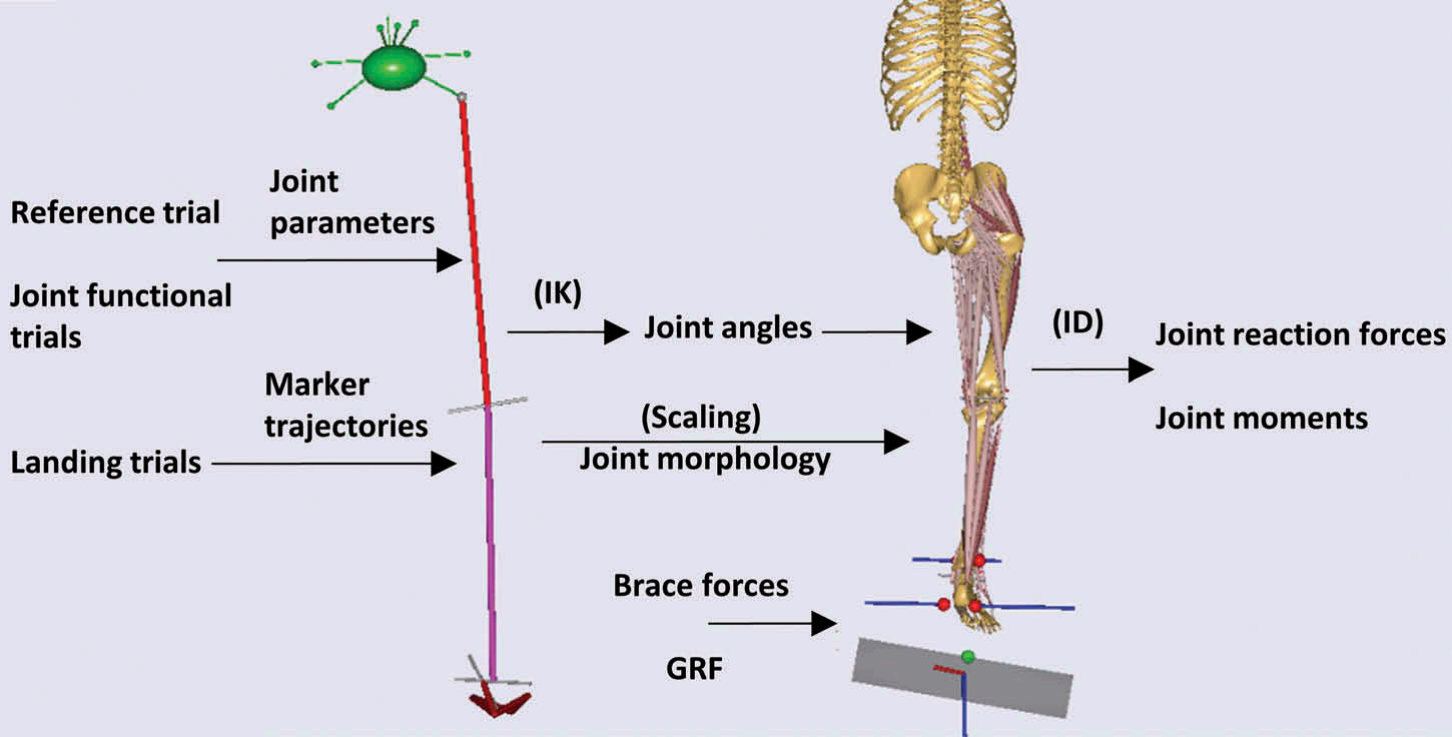
Overview of the modeling procedures followed to compute the angles, resulted in forces and moments of the subtalar and the talocrural joints from the recorded trials. IK: inverse kinematics, ID: inverse dynamics

Subsequently, the geometry of the segments as computed from the reference trial, the joints axes as defined via the optimization process, and the marker trajectories from the landing trials served as input for the kinematic analysis. The kinematic model had a total of 12 degrees of freedom (DoF), which were driven by the trajectories of the segment markers. The over-determined kinematic solver (Andersen et al. 2009) was selected to track the recorded marker trajectories in a least squares sense. Subtalar and talocrural angles were computed by the inverse kinematics analysis for all landing trials (Figure 3).

The talus coordinate system was defined at the standing reference trial:O: The origin coincident with midpoint of the malleoli.Z: The line connecting the malleoli, and pointing to the right.X: The line perpendicular to the torsional plane of the tibia/fibula, and pointing anteriorly.Y: The common line perpendicular to X-and Z-axis.JCS and motion for the talocrural jointe1: The axis fixed to the tibia/fibula and coincident with the Z-axis of the tibia/fibula coordinate system.Rotation (a): dorsiflexion (positive) or plantarflexion (negative).Displacement (q1): medial (negative) or lateral (positive) shift.e3: The axis fixed to the talus and coincident with the y-axis of the talus coordinate system.Rotation (g): internal rotation (positive) or external rotation (negative).Displacement (q3): correspond to compression (positive) or distraction (negative).e2: The floating axis, the common axis perpendicular to e1 and e3.Rotation (b): inversion (positive) or eversion (negative).Displacement (q2): anterior (positive) or posterior (negative) drawer.JCS and motion for the subtalar jointe1: The axis fixed to the talus and coincident with the Z-axis of the talus coordinate system.Rotation (a): dorsiflexion (positive) or plantarflexion (negative).Displacement (q1): medial (negative) or lateral (positive) shift.e3: The axis fixed to the foot and coincident with the y-axis of the foot coordinate system.Rotation (g): internal rotation (positive) or external rotation (negative).Displacement (q3): correspond to compression (positive) or distraction (negative).e2: The floating axis, the common axis perpendicular to e1 and e3.Rotation (b): inversion (positive) or eversion (negative).Displacement (q2): anterior (positive) or posterior (negative) drawer.

#### 2.2.2. Musculoskeletal model

A musculoskeletal model was employed for the inverse dynamics analysis. Measurements of a cadaver specimen (Klein Horsman et al. 2007) were used as a generic template of each individual’s anatomy. Anatomical landmarks and joints parameters of the musculoskeletal model were mapped to the corresponding parameters of the stick figure model, to ensure that the scaling transformation of the musculoskeletal model will match the kinematics of stick-figure model (for more details see Lund et al. (2015)). A constant strength muscle model was used, and the min/max criterion (Damsgaard et al. 2006) served to solve the muscle recruitment problem in the inverse dynamics analysis.

Joint angles, as computed by the kinematic analysis, COP and the net forces from the force plate measurements and brace pressure data were implemented into the musculoskeletal model. Two reference frames on each malleoli were used to implement the brace reaction forces into the model. The first reference frame had the orientation of the foot segment, whereas the second reference frame had the orientation of the shank segment. The COP coordinates from each area were projected into the respective reference frames, and the summed force was assumed to act perpendicular to the sagittal plane within each segment.

Inverse dynamics analyses were performed for all trials, in order to compute joint reaction forces and moments (Figure 3). For the braced trials, the inverse dynamics analysis was performed with and without the implementation of the brace reaction forces to assess the contribution of the brace pressure to the computed variables.

### 2.3. Data analysis

For each trial, peak magnitudes of the GRF components were identified and normalized to body weight (N/BW). Furthermore, angles, reaction forces, and moments were computed for the subtalar and talocrural joints. The subtalar kinetic variables were computed with respect to the calcaneus reference frame while the talocrural kinetic variables were computed with respect to the shank reference frame. The joints’ ROM were expressed in degrees (°) and computed over three time periods: 200 ms before the initial contact of the foot with the platform (PRE), 50 ms after the IC, defined as early contact (ECO), and 50–200 ms after IC, defined as late contact (LCO). Additionally, the joint angles at IC were computed. Reaction forces expressed in N/BW and moments expressed in Nm/BW were computed for each joint over ECO and LCO.

Two independent variables were tested. The brace factor had two levels (braced and unbraced condition), and the inclination factor had three levels (−5°, 0°, and 15°). Six 2 × 3 repeated measures MANOVAs were performed to assess the effect of the ankle brace and the surface alignment on GRF (1), on kinematic variables (2), on joint reaction forces during ECO (3) and LCO (4), on the joint moments during ECO (5) and LCO (6) (Table 1). When significant differences were observed, separate two-way (2 × 3) repeated measures ANOVA tested for group differences. The mean values of the respective dependent variables for six successive repetitions per group were used for each test. When the sphericity assumption was violated, the degrees of freedom were corrected using the Greenhouse-Geisser estimates of sphericity. When significant differences were identified for the inclination factor, paired *t*-tests compared all pairs of levels with the significance value being adjusted using a Bonferroni correction. A commercially available statistical analysis package SPSS v.20 (IBM Corp®, USA) was used. Since six separate MANOVA tests were employed for the statistical analysis, a more conservative significance value of *P* < 0.01 was chosen for all analyses.

**Table 1.** Groups of dependent variables

Group	Name	Variables
1	GRF	Peak magnitude of vertical GRF, Peak magnitude of mediolateral GRF, Peak magnitude of anterioposterior GRF
2	Kinematics	Talocrural ROM PRE, Talocrural ROM ECO, Talocrural ROM LCO, Subtalar ROM PRE, Subtalar ROM ECO, Subtalar ROM LCO, Talocrural plantarflexion IC, Subtalar inversion IC
3	Forces ECO	Talocrural compression ECO, Talocrural mediolateral ECO, Talocrural anterioposterior ECO, Subtalar compression ECO, Subtalar mediolateral ECO, Subtalar anterioposterior ECO
4	Forces LCO	Talocrural compression LCO, Talocrural mediolateral LCO, Talocrural anterioposterior LCO, Subtalar compression LCO, Subtalar mediolateral LCO, Subtalar anterioposterior LCO
5	Moments ECO	Talocrural dorsiflexion ECO, Talocrural inversion ECO, Talocrural internal rotation ECO, Subtalar dorsiflexion ECO, Subtalar inversion ECO, Subtalar internal rotation ECO
6	Moments LCO	Talocrural dorsiflexion LCO, Talocrural inversion LCO, Talocrural internal rotation LCO, Subtalar dorsiflexion LCO, Subtalar inversion LCO, Subtalar internal rotation LCO

Abbreviations: GRF: ground reaction forces, ROM: range of motion, ECO: early contact, LCO: late contact, PRE: before the initial contact, IC: initial contact.

## 3. Results

No significant interaction between the brace and inclination factors was observed for any of the tested groups.

### 3.1. Brace factor

The brace factor had a significant effect on the kinematic variables (*P* < 0.001) and the forces during ECO (*P* = 0.001) (Table 2). Univariate tests (Table 3) showed that participants landed with a 12.4° significantly less plantarflexed, and a 4.6° significantly more everted foot orientation for the braced condition. Furthermore, the subtalar ROM was significantly reduced during PRE (*P* = 0.001) and ECO (*P* = 0.002), while the talocrural ROM was significantly reduced during ECO (*P* < 0.001) and LCO (*P* < 0.001). A significantly increased subtalar compression force was observed for the brace condition during ECO (*P* = 0.009).

**Table 2.** Summary of MANOVA results

Factor		Variable	Wilks Λ	F value	P-value	Partial η^2^
Brace	GRF		0.783	1.297	0.314	0.217
	Kinematics*		0.068	15.407	< 0.001	0.932
	Forces ECO*		0.181	8.270	0.001	0.819
	Forces LCO		0.681	0.858	0.553	0.319
	Moments ECO		0.451	2.231	0.118	0.549
	Moments LCO		0.593	1.259	0.350	0.407
Inclination	GRF*		0.031	47.046	< 0.001	0.825
	Kinematics*		0.300	2.577	0.005	0.452
	Forces ECO		0.621	1.210	0.301	0.212
	Forces LCO*		0.322	3.430	0.001	0.433
	Moments ECO*		0.222	5.043	< 0.001	0.528
	Moments LCO*		0.135	7.751	< 0.001	0.633

* P < 0.01 Abbreviations: GRF: ground reaction forces, ECO: early contact, LCO: late contact.

**Table 3.** Means and standard deviations (SD) of the significantly different variables for the brace factor

Variable	NB Mean SD		WB Mean SD		F value	P value	Partial η^2^
Subtalar compression force ECO (N/BW)	8.43	3.65	9.83	3.85	8.827	0.009	0.356
Subtalar ROM PRE (°)	6.6	3.6	4.6	2.9	16.372	0.001	0.506
Talocrural plantarflexion angle IC (°)	23.5	11.8	11.1	12.7	46.914	< 0.001	0.746
Subtalar eversion angle IC (°)	4.7	7.8	9.2	7.4	15.866	0.001	0.498
Talocrural ROM ECO (°)	18.7	5.6	10.7	5.4	46.115	< 0.001	0.742
Subtalar ROM ECO (°)	8.7	5.3	6.1	4.2	14.084	0.002	0.468
Talocrural ROM LCO (°)	17.5	5.8	11.9	4.6	50.345	< 0.001	0.759

Abbreviations: NB: no brace, WB: with brace, ROM: range of motion, ECO: early contact, LCO: late contact, PRE: before the initial contact, IC: initial contact.

### 3.2. Inclination factor

The inclination factor (Table 2) showed significant effects on the GRF (*P* < 0.001), the kinematic variables (*P* = 0.005), the joint forces during LCO (*P* = 0.001) and the joint moments during ECO (*P* < 0.001) and during LCO (*P* < 0.001). For the GRF, univariate tests (Table 4) revealed significant differences for the peak magnitudes of the mediolateral (*P* < 0.001) and vertical (*P* < 0.001) components. Both the peak magnitudes of the mediolateral and the vertical GRF were higher when participants landed on the inverted surface compared to neutral.

**Table 4.** Means and standard deviations (SD) of the significantly different variables for the brace factor

Variable	Eversion Mean SD		Neutral Mean SD		Inversion Mean SD		F value	P value	Partial η^2^
Peak vertical GRF (N/BW)^bc^	3.76	0.90	4.06	0.87	4.26	0.98	21.883	< 0.001	0.578
Peak mediolateral GRF (N/BW)^bc^	−0.03	0.02	0.02	0.07	1.38	0.41	301.345	< 0.001	0.950
Subtalar compression LCO (N/BW)^b^	8.69	3.52	9.30	3.77	8.18	2.96	4.307	0.022	0.212
Talocrural inversion ECO (Nm/BW)^abc^	0.01	0.11	0.05	0.08	0.08	0.08	15.655	0.001	0.495
Talocrural internal rotation ECO (Nm/BW)^a^	0.02	0.17	−0.03	0.13	−0.05	0.14	9.296	0.004	0.367
Subtalar internal rotation ECO (Nm/BW)^ac^	−0.01	0.14	0.03	0.11	0.06	0.12	9.311	0.003	0.368
Talocrural inversion LCO (Nm/BW)^ac^	0.02	0.09	0.07	0.07	0.08	0.07	16.407	< 0.001	0.506
Talocrural internal rotation LCO (Nm/BW)^ac^	−0.02	0.12	−0.07	0.10	−0.07	0.10	21.007	< 0.001	0.568
Subtalar internal rotation LCO (Nm/BW)^ac^	0.01	0.10	0.07	0.09	0.06	0.09	16.365	< 0.001	0.506
Talocrural ROM ECO (°)^ac^	13.4	6.5	14.7	6.5	15.9	7.03	11.445	0.001	0.417
Subtalar ROM ECO (°)^a^	8.4	5.3	7.5	4.9	6.4	4.3	7.057	0.012	0.306
Talocrural ROM LCO (°)	14.1	5.8	14.4	6.0	15.5	6.0	6.111	0.006	0.276

a: denotes significant difference between everted and neutral surfaces. b: denotes significant difference between inverted and neutral surfaces. c: denotes significant difference between everted and inverted surfaces. Abbreviations: ROM: range of motion, ECO: early contact, LCO: late contact, PRE: before the initial contact, IC: initial contact.

For the kinematic variables, the inclination factor had an effect on the subtalar ROM during ECO (*P* = 0.012) and the talocrural ROM during ECO (*P* = 0.001) and LCO (*P* = 0.006). The talocrural ROM during ECO was increased by 1.2° for the inverted, and it was decreased by 1.3° for the everted alignment in comparison to the neutral one. The subtalar compression force during LCO was decreased by 1.12 N/BW for the inverted surface compared to the neutral surface alignment (*P* = 0.022).

Regarding the joint moments during ECO, significant differences were observed for the talocrural inversion moment (*P* = 0.001), the talocrural internal rotation moment (*P* = 0.004), and the subtalar internal rotation moment during ECO (*P* = 0.003). Post hoc analysis revealed that the talocrural inversion moment was increased by 0.02 Nm/BW for the inverted and decreased by 0.04 Nm/BW for the everted compared to the neutral surface alignment. The subtalar internal rotation moment was decreased by 0.02 Nm/BW for the everted compared to the neutral surface alignment.

Regarding the joint moments during LCO, the subtalar internal rotation moment was significantly decreased by 0.05 Nm/BW for the everted compared to the neutral surface alignment (*P* < 0.001). The talocrural inversion moment (*P* < 0.001) and external moment during (*P* < 0.001) were 0.05 Nm/BW and 0.06 Nm/BW, respectively, lower for the everted surface compared to the neutral.

## 4. Discussion

### 4.1. Brace effects

Our hypothesis of similar peak magnitudes of the GRF was supported. Our results are in agreement with the results presented by Cordova et al. (2010) that also employed a semi-rigid ankle brace for their study. On the other hand, Niu et al. (2014) hypothesized that semi-rigid ankle braces would increase the impact force, due to the imposed kinematic changes on the ankle joint, to alter the energy absorption by the free motion of the ankle joint. A possible explanation for the observed similar peak magnitudes of the GRF could be that semi-rigid braces also affect knee kinematics by resulting in an altered landing orientation (DiStefano et al. 2008; Simpson et al. 2013), which potentially increases the energy absorption by the knee (Devita and Skelly 1992).

As hypothesized, bracing restricted the ROM of the ankle joint complex. Prior to landing, the subtalar ROM was reduced for the braced condition. Our results support the hypothesis that bracing influences the position of an unloaded foot prior to IC, by decreasing the ankle ROM (Eils and Rosenbaum 2003). As the subtalar joint is mainly responsible for foot in- and eversion, the prophylactic function of the brace of restricting foot inversion was confirmed. This outcome has important clinical relevance since the foot orientation during the flight phase can be important for avoiding ankle injuries (Eils and Rosenbaum 2003). The restriction of the subtalar ROM prior to landing prevents a landing orientation with excessive foot inversion, which is considered as one potential injury mechanism of lateral ankle sprains.

As expected, the reduced subtalar ROM prior to landing resulted in an altered positioning at IC. As it was shown before (McCaw and Cerullo 1999; Cordova et al. 2010) bracing restricted ankle inversion and plantarflexion, forcing participants to land with a less plantarflexed and more everted foot orientation. Such foot positioning has been suggested to reduce the moment arm of the GRF vector about the subtalar axis (Wright et al. 2000).

During the contact phase, bracing restricted ankle joint ROM in both the frontal and sagittal planes. This outcome was anticipated since previous studies have shown that some rigid ankle braces reduce both inversion and plantarflexion (McCaw and Cerullo 1999; Cordova et al. 2010). The latter suggests that some ankle braces restrict the shank motion not only in the frontal but in the sagittal plane as well.

The reduced subtalar ROM and the landing positioning can be considered as a positive outcome since a common lateral ankle sprain mechanism is due to excessive foot inversion. However, the talocrural ROM reduction combined with the reduced plantarflexion at IC might have negative effects on knee loading. As ankle plantarflexion is essential for energy absorption (Devita and Skelly 1992), a reduction of the normal motion of the ankle complex in the sagittal plane may result in adverse effects on the knee or/and the hip. Although some studies investigated the effects of ankle bracing on the knee (Venesky et al. 2006; DiStefano et al. 2008; Simpson et al. 2013; Vanwanseele et al. 2014), more research is needed to establish whether ankle bracing influences knee injury rates (Dizon and Reyes 2010).

Our expectation for differences in the joints kinetics was partially supported. A significantly higher subtalar compression force was observed for the braced condition during the early contact. This higher force may increase the load on individual structures within or around the ankle joint complex, such as articular cartilage or ligaments. Although prophylactic ankle bracing over a whole playing season has been recommended (DiStefano et al. 2008), an increased subtalar compression force combined with long-term use might contribute to overuse injuries. A more detailed model, which entails ligamentous structures and contact forces of the articular surfaces, could provide more information regarding the distribution of the joint reaction forces and moments to the individual joint structures such as ligaments and articular surfaces. Such a model could be used to compute the loading to individual structures among different brace conditions and therefore estimate potential complications from long-term use of ankle braces by assessing possible excessive ligament or articular contact loading.

On the other hand, no differences were observed for the joint moments. Although, it could be expected that the observed kinematic differences would result in alterations in the joint moments, no significant differences were found, possibly due to individual alterations from landing to landing. This is in contrast with the suggestion that the observed landing positioning for the brace condition would reduce the moment arm of the GRF vector about the subtalar axis (Wright et al. 2000) in the tested landing situation.

### 4.2. Inclination effects

No differences were observed in ankle joint kinematics prior to and at initial contact. This outcome indicates that our experimental protocol provided the same visual/audible conditions among the different surface alignments. The participants followed the researcher’s instructions and performed the landing task, without looking at the platform. A potential prior knowledge of the surface inclination could lead participants to prepare differently to varying alignments, which could possibly result in kinematic adjustments for the different surface alignments. It should be noted, however, that a general anticipation effect might be present. While we are confident that the participants were not able to anticipate which inclination of the landing surface was present in each trial, they may have stiffened their ankles by co-contracting the muscles of the lower leg in anticipation of the different landing conditions. Such could potentially explain the same ankle kinematics prior to initial contact.

As expected, some kinematic differences were observed during the contact phase. The different surface alignments lead to different foot positioning on the platform, which resulted in kinematic differences in the ankle joint complex in accordance with the direction of the platform tilt. These different foot positions among the surface alignments led to the alterations in the ROM of the ankle joint complex during the contact phase. However, these differences were substantially smaller than the platform inclinations (~ 1°), which is without clinical relevance but indicates the role of the muscles in actively stabilizing the joints after making contact.

In contrast to the brace factor, significant differences were observed in the peak magnitudes of the GRF components for the inclination factor. The different surface alignments caused different foot alignments, leading to higher peak magnitudes for the vertical and the mediolateral GRF components for the inverted surface. A possible explanation for this could be that the subjects did not compensate for the altered ankle kinematics by knee joint excursion to absorb the impact energy, resulting in an increase in the GRF. These kinematic differences also led to alterations in ankle kinetics. During landing on the everted surface, the talocrural inversion and internal rotation moments, and the subtalar internal rotation moment were reduced compared to landing on the neutral surface. These reductions are likely caused by changes in the CoP location when participants landed on the everted surface. Regarding the inverted alignment, the significantly higher talocrural inversion moment can be attributed to the significantly higher peak magnitudes of GRF components, possibly in conjunction with an altered CoP.

Clinically, the latter outcomes may explain why landing on irregular surfaces, i.e. another player’s foot or a rutted field, is considered a risk factor for ankle sprains (Gross and Liu 2003). While landing on inclined surfaces, ligamentous structures surrounding the ankle joint complex have to absorb higher forces and moments, which potentially increase the risk of ligamentous damage. Furthermore, the higher talocrural inversion moment during landing on the inverted surface combined with the higher peak magnitudes of the vertical and the mediolateral GRF indicates the task being more challenging which, potentially increases the injury risk for the lateral ankle ligaments (Renstrom and Konradsen 1997).

### 4.3. Brace reaction forces

The brace reaction forces were measured with a pressure system, and these were included in the computational model, which is a new method not been implemented in previous studies. However, the inclusion of these forces resulted in small differences in the mean values of the kinetic variables during ECO and LCO. Such small force contributions are most likely the cause for the kinematic adjustments prior to IC. It has to be noted that there were considerable differences in individual subjects (Suppl. Tables 5 and 6). Therefore, this new method could potentially be used to vary the geometry of an ankle brace in a subject specific manner to achieve an optimal foot placement at IC. During contact, the brace reaction forces remained small in relation to external GRF in the landing conditions recorded within this experiment. While introducing an unexpected landing configuration, the conditions were still safe by not forcing the ankle joints beyond their anatomical ranges of motion. The brace forces will most likely increase substantially in a situation where the ankle is forced beyond its range of motion. The model could then be used to estimate brace contributions in critical loading situations.

**Table 5.** Differences of joint reaction forces as calculated with and without the implementation of the brace reaction forces. The highest three values per variable and the mean value are presented

			Variable difference	
Period	Joint	Variable	Highest values (N/BW)	Mean value (N/BW)
ECO	Talocrural	Medial	0.56	0.024
			0.64	
			0.95	
		Compression	0.85	0.004
			−2.22	
			2.37	
		Anterior	0.27	−0.003
			0.52	
			−0.75	
	Subtalar	Medial	−2.02	−0.174
			−2.25	
			−6.08	
		Compression	−2.32	0.154
			−3.13	
			9.03	
		Anterior	−2.32	0.085
			3.25	
			3.43	
LCO	Talocrural	Medial	−0.41	0.004
			0.64	
			−0.65	
		Compression	−0.98	0.004
			−1.04	
			1.62	
		Anterior	−0.75	0.040
			0.78	
			1.74	
	Subtalar	Medial	−3.02	−0.041
			−3.11	
			4.18	
		Compression	−4.64	−0.264
			4.66	
			−4.98	
		Anterior	−2.19	−0.069
			2.70	
			−2.74

**Table 6.** Differences of joint moments as calculated with and without the implementation of the brace reaction forces. The highest three values per variable and the mean value are presented

			Variable difference	
Period	Joint	Variable	Highest values (Nm/BW)	Mean value (Nm/BW)
ECO	Talocrural	Inversion	0.21	0.010
			0.23	
			0.38	
		Internal rotation	−0.03	−0.002
			−0.10	
			−0.22	
		Plantarflexion	−0.01	0.001
			0.01	
			0.02	
	Subtalar	Dorsiflexion	0.11	0.002
			0.13	
			−0.23	
		Internal rotation	0.06	−0.002
			−0.09	
			−0.16	
		Eversion	0.03	0.001
			−0.04	
			0.05	
LCO	Talocrural	Inversion	0.40	0.007
			0.57	
			−0.62	
		Internal rotation	−0.25	0.005
			0.65	
			0.98	
		Plantarflexion	−0.40	0.000
			0.45	
			−0.47	
	Subtalar	Dorsiflexion	0.46	0.023
			0.48	
			−0.59	
		Internal rotation	−0.54	−0.008
			−0.59	
			−0.83	
		Eversion	0.17	0.009
			0.21	
			0.37	

### 4.4. Limitations

Some limitations associated with the computational model used in the study have to be considered. The foot was modeled as two rigid bodies even though the significance of a multi-rigid foot model has been highlighted (Arampatzis et al. 2002). However, the geometry of the brace did not allow for marker placement on the foot segments needed for such an approach. Furthermore, the model neglects ligamentous structures. For example, our results showed similar joint moments for the brace conditions; however, the observed kinematic differences combined with the similar GRF suggest that ligamentous structures around ankle joint complex probably were loaded differently. Thus, a more detailed computational model, which takes these ligamentous structures and articular surface geometry into account, should be developed.

Regarding the experimental procedures used in this study, it has to be highlighted that our results are limited to the specific testing population (healthy male athletes without previous ankle injury), the testing experimental procedure (single leg drop landing), and the tested ankle brace (Sports Stirrup Aircast).

## 5. Conclusions

The Sports Stirrup Aircast ankle brace altered ankle joint kinematics in both the frontal and the sagittal plane. The kinematic reductions in the frontal plane are considered as a positive outcome since it indicates that the tested ankle brace prevents excessive foot inversion. However, the kinematic reductions in the sagittal plane may result in increased knee loading. No differences in joint moments during the contact phase were revealed while a higher subtalar compression force for the braced condition was observed. Although this may have long-term consequences for ankle brace users, no predictions are possible based on the current results. During landing on the inverted surface, the increased peak magnitudes of the vertical and the mediolateral GRF components, and talocrural inversion moment compared to the neutral surface alignment, likely constitute an increased risk for lateral ankle ligaments injuries.
